# Supplementary material for: The Cerato-Platanin EPL2 from Trichoderma reesei Is Not Directly Involved in Cellulase Formation but in Cell Wall Remodeling
Source: Microorganisms. 2023 Jul 31;11(8):1965. doi: 10.3390/microorganisms11081965 (PMC10459490; doi:10.3390/microorganisms11081965)
Supplement: Supplementary file 1 [file microorganisms-11-01965-s001.zip › microorganisms-2418105-supplementary.pdf]

Supplementary Material

The Cerato-platanin EPL2 from *Trichoderma reesei* is directly involved not in cellulase formation but in cell wall remodeling

Alinne Costa Silva [†], Letícia Harumi Oshiquiri [†], Luiz Felipe de Morais Costa de Jesus, David Batista Maués and Roberto N. Silva *

Figure S1

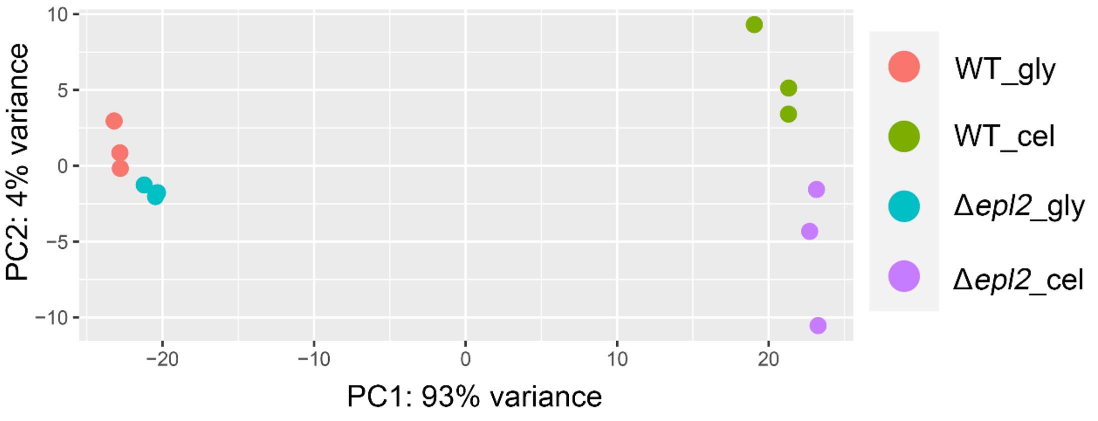

Figure S1. Principal component analysis (PCA) of the different conditions analyzed. WT represents the wild type *T. reesei* QM6aΔ*tmus53*Δ*pyr4* strain. Gly indicates that the strains were cultivated on 1% glycerol for 24 hours. Cel indicates that the strains were initially cultivated on 1% glycerol for 24 hours, then transferred to fresh media containing 1% cellulose and incubated for an additional 72 hours.

Figure S2

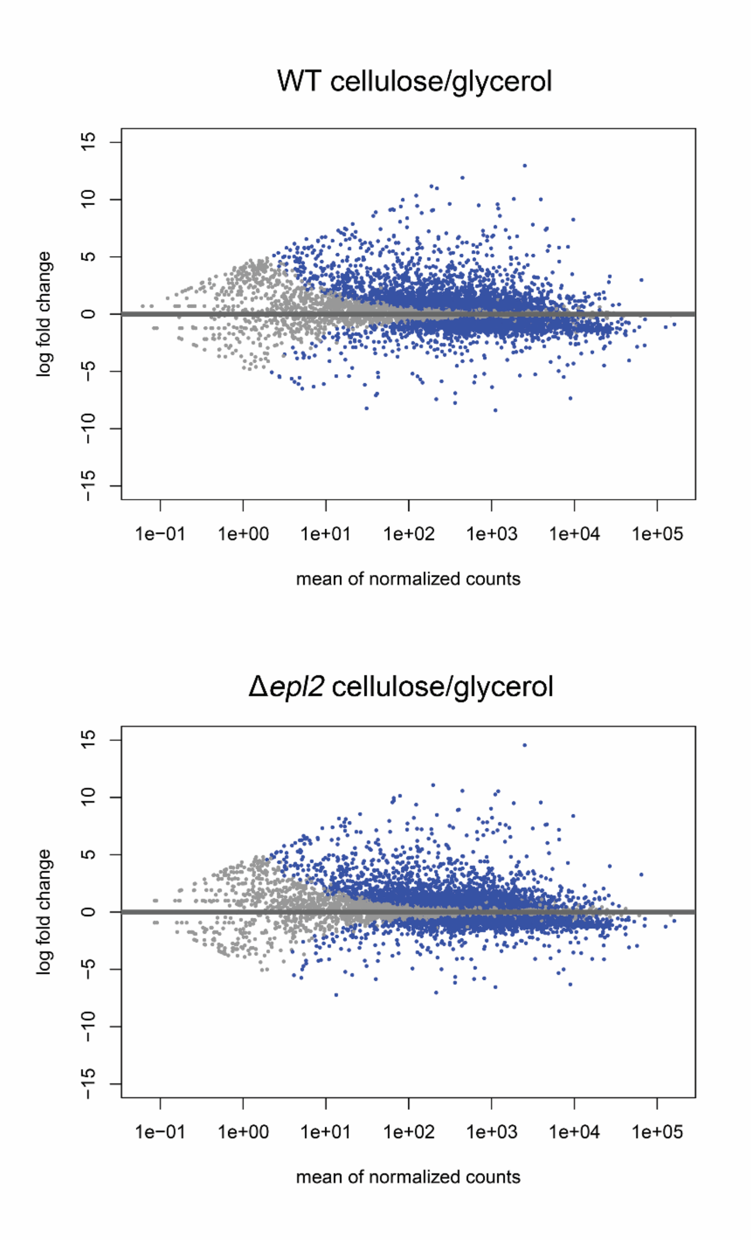

Figure S2. Differentially expressed genes in wild type (WT) and Δ*epl2* strains. The expression profile was generated for growth on 1% cellulose for 72 hours in relation to 1% glycerol for 24 hours. Genes that exhibit differential expression are represented in blue, while all other genes of the genome are depicted in gray.

Figure S3

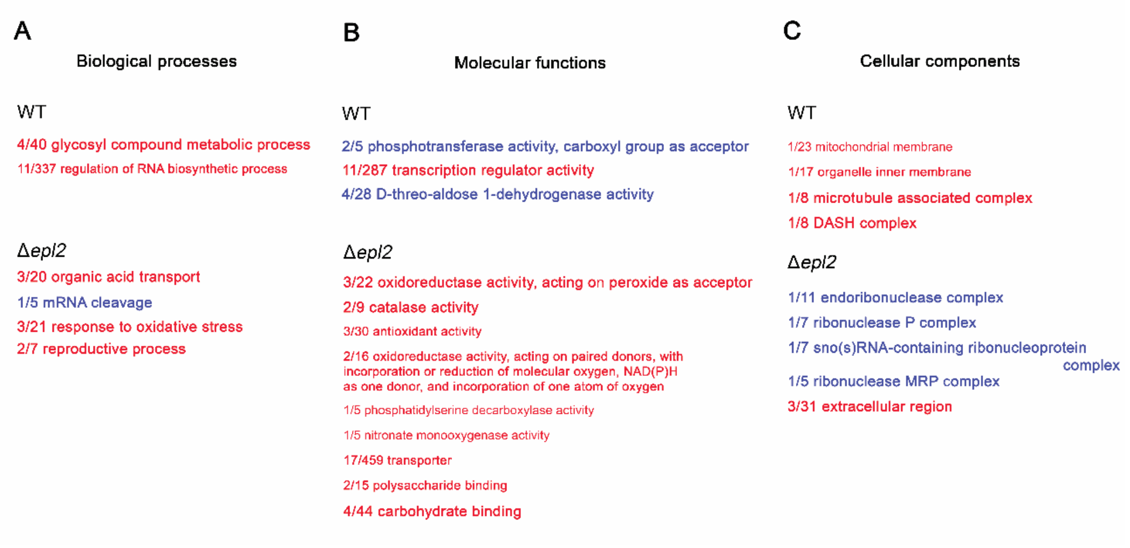

Figure S3. Gene Ontology enrichment analysis of WT and Δ*epl2* strains. Biological processes (A), Molecular functions (B), and Cellular compartments (C) involved in the growth of WT and Δ*epl2* strains on 1% cellulose compared to 1% glycerol. Upregulated processes are represented in red, while downregulated processes are shown in blue. The fractions before each category indicate the number of regulated genes relative to the total number of *T. reesei* genes in that category. All processes shown have a statistical significance of $p < 0.05$.

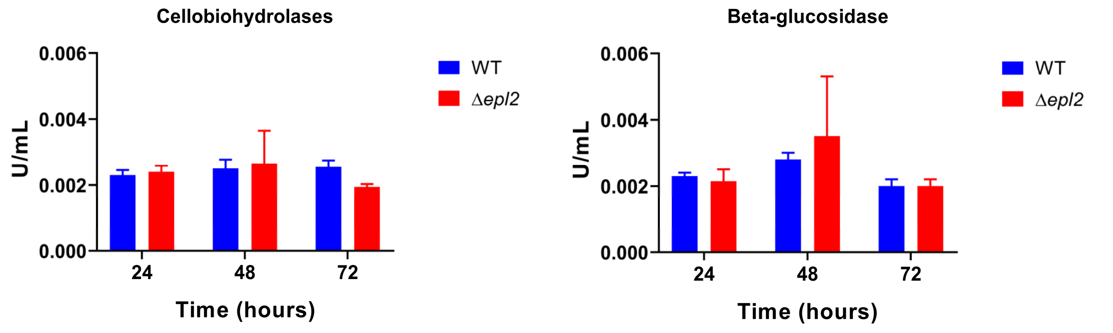

Figure S4: Enzymatic activity of WT and Δ*epl2* strains grown on cellulose. No significant changes were observed

Supplementary tables

Table S1: Proteins used to infer the cladogram.

Protein (NCBI ID, JGI ID)	Organism	Reference (DOI)
EPL1 (XP_013937770.1)<br>EPL2 (XP_013944228.1)<br>EPL3 (XP_013937568.1)	*Trichoderma atroviride*	10.1074/jbc.M112.427633<br>10.3389/fpls.2015.00077
Sm1 (XP_013959806.1)<br>Sm2 (XP_013954872.1)<br>Sm3 (XP_013960015.1)	*Trichoderma virens*	10.1094/MPMI-19-0838<br>10.3389/fpls.2015.00077<br>10.1186/s12866-014-0333-0
Epl1-Tas (JN966996)<br>Epl (XP_024761368.1)<br>Epl (XP_024761426.1)<br>Epl (XP_024760848.1)	*Trichoderma asperellum*	10.1038/s41598-018-26328-1
Epl-1 (XP_024774373.1, 508110)<br>Epl (XP_024774126.1)<br>Epl (XP_024771835.1)	*Trichoderma harzianum*	10.1038/srep17998
EPL (XP_006970000.1, 82662)<br>EPL (XP_006969009.1, 111449)<br>EPL2 (XP_006969193.1, 123955)	*Trichoderma reesei*	This study
FgCPP1 (FGSG_10212)	*Fusarium graminearum*	10.1080/07060661.2022.2044910
BcSpl1 (XP_024547455.1)<br>Bcspl2 (XP_001552257.1)	*Botrytis cinerea*	10.1111/j.1469-8137.2011.03802.x
Sscp1 (XP_001588549.1)	*Sclerotinia sclerotiorum*	10.1111/nph.14842

MSP1 (XP_359969.1)	*Magnaporthe oryzae*	10.1111/j.1574-6968.2007.00796.x
CP (EF017218)	*Ceratocystis platani*	10.1111/j.1574-6968.2011.02475.x
SP1 (AAM33130.1)	*Leptosphaeria maculans*	10.1007/s10658-005-3168-6

Table S2: Primer sequences used in this study.

Amplified fragment	Sequence
pRS426_5’ UTR *epl1* Forward	5’ GTAACGCCAGGGTTTTCCCATCGCGTCTTGGGCGGAACTT 3’
*pyr4*_5’ UTR *epl1* Reverse	5’**GCGGGAGAGTCTGGATAGCTCGAAGG**CAGGAGAAAAGCACAAAGAAGAG 3’
*pyr4*_3’ UTR *epl1* Forward	5’**GTCGACGATATCAGCTTCCATAT**CCCGTCTTATTCCCAATGTTG 3’
pRS426_3’ UTR *epl1* Reverse	5’GGAAACAGCTCATCTTCTCGTCGACAACCGCGGATAACAATTTCACACA 3’
pyr4 Forward	5’CCTTCGAGCTATCCAGACTCTCCCTTGCCTTGTCTTGTCTGGGTT 3’
pyr4 Reverse	5’CAACATTGGGAATAAGACGGGCGGTTGATTGTTGCCGTCCGTTTC 3’
epl1 Forward	5’ CAACTCGCCCAATTGTTGACGG 3’
epl1 Reverse	5’ GGTGTCGGGGAAAACAGAGA 3’

Table S3: Primers used in RT-qPCR experiments.

Protein ID	Name	5’ → 3’ (Forward)	3’ → 5’ (Reverse)
123955	epl1	CGGTACAACTGCCACTCG	GAGCACGATACACTAGCCAAG
73643	beta-1,6-N-acetylglucosaminyltransferase	GCCACAAGAATGCTACGAATG	GTCCAGGGATGATGTAGTTGAG
69700	alpha-N-acetylglucosaminidase	CGCATGGTTGAGCTAGGTATAA	GGAGAGGCCAAATCCAGAAA
71532	glucan endo-1,3-alpha-glucosidase	CCGATCTCGTGGAACTCATTAC	GCCCTGTTGGTAGTTCTGTATC
59791	Chitinase	GATTCAACAACACGGCTACAAC	TCCCAAGCCTGTCCTACTAA
79671	N-acetyl-glucosamine-6-phosphate deacetylase	ACATCCTACAATCCCACCATTAC	CGTCTGTTTCTCGGACAAGAT

123639	Glycoside Hydrolase Family 64	CCCTCTCTCATCACAAG AGTT	GACAGCCTTTCCTTCGG TATAG
123726	Glycoside Hydrolase Family 16	GCAACCTCAACGTCAAC AAC	GGAACCAGTGATCTGGA AGAAG
108672	glucan endo-1,3-alpha-glucosidase	GTCCGGTGTTAACCCTT ACTT	GACGTGCTCATCCTCGA TATAC
54242	Glycoside Hydrolase Family 55	ACAGCGGCTTCACCATT AG	GGACCAGAAGTTGTCGA TGTAG
72567	Cel6A	ACAAGAATGCATCGTCT CCG	TGTTCCACCCGTTGTAG TTG
123989	Cel7a	CCGAGCTTGGTAGTTAC TCTG	GGTAGCCTTCTTGACTG AGT
49081	Cel74a	CGGTTGTATCGTACCTA TTCCG	TGATGTCTTTCCAAGTT CCCC
76672	Cel3a	CTGTACATCACCTACCC ATC	TAGCTGAGATCTCGTCG TC

Table S4: Summary of reads obtained by RNA Sequencing

Sample	Raw Reads	Mapped Reads	% Mapped Reads
WT Glycerol 1	14.925.755	14.633.646	98.04
WT Glycerol 2	14.333.019	14.109.929	98.44
WT Glycerol 3	15.515.433	15.291.320	98.56
Δ*epl2* Glycerol 1	16.542.757	16.309.899	98.59
Δ*epl2* Glycerol 2	14.657.347	14.463.704	98.68
Δ*epl2* Glycerol 3	15.545.576	15.310.844	98.49
WT Cellulose 1	16.106.435	15.870.387	98.53
WT Cellulose 2	12.982.812	12.778.157	98.42
WT Cellulose 3	13.774.576	13.587.729	98.64
Δ*epl2* Cellulose 1	12.829.839	12.643.452	98.55
Δ*epl2* Cellulose 2	12.761.500	12.572.948	98.52
Δ*epl2* Cellulose 3	14.061.109	13.861.279	98.58
